# Towards achieving the family planning targets in the African region: a rapid review of task sharing policies

**DOI:** 10.1186/s12978-020-01038-y

**Published:** 2021-01-23

**Authors:** Leopold Ouedraogo, Desire Habonimana, Triphonie Nkurunziza, Asmani Chilanga, Elamin Hayfa, Tall Fatim, Nancy Kidula, Ghislaine Conombo, Assumpta Muriithi, Pamela Onyiah

**Affiliations:** 1 Reproductive, Maternal Health and Ageing Team, World Health Organization, Regional Office for Africa, Brazzaville, Republic of the Congo; 2 Department of Community Medicine, Research and Innovation Unit, Faculty of Medicine, University of Burundi, Bujumbura, Burundi; 3 Reproductive, Maternal Health and Ageing Team, Intercountry Support Team for West Africa, World Health Organization, Ouagadougou, Burkina Faso; 4 Reproductive, Maternal Health and Ageing Team, Intercountry Support Team for East and Southern Africa, World Health Organization, Harare, Zimbabwe; 5 Reproductive, Maternal Health and Ageing Team, Intercountry Support Team for Central Africa, World Health Organization, Libreville, Gabon

**Keywords:** Family planning, Task sharing, World health organisation, African region, planification familiale, partage des tâches, Organisation mondiale de la Santé, Région africaine

## Abstract

**Background:**

Expanding access and use of effective contraception is important in achieving universal access to reproductive healthcare services, especially in low- and middle-income countries (LMICs), such as those in sub-Saharan Africa (SSA). Shortage of trained healthcare providers is an important contributor to increased unmet need for contraception in SSA. The World Health Organization (WHO) recommends task sharing as an important strategy to improve access to sexual and reproductive healthcare services by addressing shortage of healthcare providers. This study explores the status, successes, challenges and impacts of the implementation of task sharing for family planning in five SSA countries. This evidence is aimed at promoting the implementation and scale-up of task sharing programmes in SSA countries by WHO.

**Methodology and findings:**

We employed a rapid programme review (RPR) methodology to generate evidence on task sharing for family planning programmes from five SSA countries namely, Burkina Faso, Cote d’Ivoire, Ethiopia, Ghana, and Nigeria. This involved a desk review of country task sharing policy documents, implementation plans and guidelines, annual sexual and reproductive health programme reports, WHO regional meeting reports on task sharing for family planning; and information from key informants on country background, intervention packages, impact, enablers, challenges and ways forward on task sharing for family planning. The findings indicate mainly the involvement of community health workers, midwives and nurses in the task sharing programmes with training in provision of contraceptive pills and long-acting reversible contraceptives (LARC). Results indicate an increase in family planning indicators during the task shifting implementation period. For instance, injectable contraceptive use increased more than threefold within six months in Burkina Faso; contraceptive prevalence rate doubled with declines in total fertility and unmet need for contraception in Ethiopia; and uptake of LARC increased in Ghana and Nigeria. Some barriers to successful implementation include poor retention of lower cadre providers, inadequate documentation, and poor data systems.

**Conclusions:**

Task sharing plays a role in increasing contraceptive uptake and holds promise in promoting universal access to family planning in the SSA region. Evidence from this RPR is helpful in elaborating country policies and scale-up of task sharing for family planning programmes.

## RESUME

**Introduction:**

L'élargissement de l'accès et de l'utilisation d’une contraception efficace est important pour parvenir à l'accès universel aux services de santé reproductive, en particulier dans les pays à revenu faible et intermédiaire, comme ceux de l'Afrique subsaharienne. L’insuffisance de prestataires de soins de santé qualifiés est un facteur important de l'augmentation des besoins non satisfaits en matière de contraception en Afrique subsaharienne. L'Organisation mondiale de la Santé (OMS) recommande le partage des tâches comme stratégie importante pour améliorer l'accès aux services de santé sexuelle et reproductive en s’attaquant à la pénurie des prestataires de soins de santé. Cette étude explore l'état des lieux, les réussites, les défis et les impacts de la mise en œuvre du partage des tâches pour la planification familiale dans cinq pays d'Afrique subsaharienne. Ces données factuelles visent à promouvoir la mise en œuvre et l'extension des programmes de partage des tâches dans les pays d'Afrique sub-saharienne par l'OMS.

**Méthodologie et résultats:**

Nous avons utilisé la méthodologie de la revue rapide des programmes (RPR) pour générer des données sur le partage des tâches pour les programmes de planification familiale de cinq pays d'Afrique subsaharienne, à savoir le Burkina Faso, la Côte d'Ivoire, l'Éthiopie, le Ghana et le Nigéria. Cela impliquait la revue documentaire des documents de politique nationale de partage des tâches, des plans de mise en œuvre et des directives, des rapports annuels sur les programmes de santé sexuelle et reproductive, des rapports des réunions régionales de l'OMS sur le partage des tâches pour la planification familiale; et des informations provenant des informateurs clés sur le contexte du pays, les programmes d'intervention, l'impact, les catalyseurs, les défis et les voies à suivre pour le partage des tâches pour la planification familiale. Les résultats indiquent principalement l'implication des agents de santé communautaires, des sages-femmes et des infirmières dans les programmes de partage des tâches avec une formation liée à l’approvisionnement de pilules contraceptives et de contraceptifs réversibles à longue durée d’action (LARC). Les résultats indiquent une augmentation des indicateurs de planification familiale pendant la période de mise en œuvre du partage des tâches. Par exemple, l'utilisation des contraceptifs injectables a plus que triplé en six mois au Burkina Faso; le taux de prévalence de la contraception a doublé avec une baisse de la fécondité totale et des besoins non satisfaits en matière de contraception en Éthiopie; et l'adoption du LARC a augmenté au Ghana et au Nigéria. Certains obstacles à la réussite de la mise en œuvre comprennent une faible rétention des prestataires de niveau inférieur, une documentation inadéquate et des systèmes peu performants de gestion des données.

**Conclusions:**

Le partage des tâches joue un rôle important dans l'augmentation de l'utilisation de la contraception et dans la promotion de l'accès universel à la planification familiale dans la région Afrique subsaharienne. Les données de ce RPR sont utiles pour l'élaboration des politiques nationales et l'intensification du partage des tâches pour les programmes de planification familiale.

**Plain English summary:**

Correct and consistent use of contraceptives has been shown to reduce pregnancy and childbirth related maternal deaths and generally improve reproductive health. However, statistics show that many women of reproductive age in SSA who ought to be using contraceptives are not using them. As a result, high rates of maternal deaths from pregnancy or childbirth-related complications have been recorded in the region. One of the key barriers to accessing family planning in SSA is the shortage of healthcare providers. To address this problem, WHO recommends task sharing as an intervention to improve access and use of sexual and reproductive health services including family planning. While task sharing guidelines have been developed and disseminated in many SSA countries, limited evidence exists on their adoption, implementation and outcomes to promote scale-up. This study undertook a rapid programme review of evidence from policy documents, implementation plans and guidelines, annual sexual and reproductive health programme reports, regional meeting reports and key stakeholder reports on task sharing to explore the status, successes, challenges and impacts of the implementation of task sharing for family planning in five SSA countries: Burkina Faso, Cote d’Ivoire, Ethiopia, Ghana, and Nigeria. We found that task sharing programmes mainly involved community health workers, midwives and nurses. The intervention led to increased modern contraception access and use and general improvement in family planning indicators during the implementation periods. Some barriers to successful implementation of task sharing include poor retention of lower cadre providers, inadequate documentation, and poor data systems.

## Background

The World Bank projects a ten-fold increase in the population of sub-Saharan Africa (SSA) between 1960 and 2050, reaching 9.7 billion people in 2050 [1]. This escalation indicates Africa’s growing fertility rate [2]. Notably, while the global fertility rate between 1990 and 2019 fell from 3.2 to 2.5 births per woman, this indicator only dropped from 6.3 to 4.6 births per woman for SSA [2]. Evidently, other regions have recorded much higher declines compared to SSA (from 4.5 to 3.4 in Oceania, from 4.4 to 2.9 in Northern Africa and Western Asia, from 3.3 to 2.0 in Latin America and the Caribbean, and from 2.5 to 1.8 in Eastern and South-Eastern Asia) [2]. This decline in fertility rate continues to occur at a much slower pace in SSA as compared to the rest of the world. In other words, while it took 19 years for fertility rates in Northern Africa and Western Asia to drop from 6 to 4 births per woman (1974 to 1993), a similar decline is expected to materialise after 34 years (1995 to 2029) in SSA [2]. With weak health systems present in fragile economies, the higher fertility rates present greater risks of unpropitious pregnancy outcomes in SSA countries [3–5].

In the light of the evidence above, a wealth of literature has established a correlation between higher fertility rates, poverty and pregnancy-related deaths/complications. For instance, of some 830 women who die daily from pregnancy or childbirth-related complications around the world, 99% of such deaths occur in low-income and middle-income countries (LMICs) [6]. It is also estimated that of the 2.6 million stillbirths that occurred globally in 2015, 98% were in LMICs [7]. Furthermore, the risk of a woman in a LMIC dying from a maternal-related cause during her lifetime is about 33 times higher compared to her counterpart in a high-income country [8]. Fortunately, interventions such as modern contraception which space and limit pregnancies significantly improve the overall health of women of reproductive age [9]. Although this remains true, SSA continues to register higher proportions of unmet contraception expectations to date [10, 11].

In SSA, 16% of women of reproductive age who desire to either terminate or postpone childbearing do not currently use a contraceptive method [12]. Most importantly, in this region, the rate of unmet needs for family planning is about 21% among married women or those living in union [12]. Such trends represent barriers to the achievement of universal access to sexual and reproductive healthcare services including for family planning by 2030 in SSA, as stipulated in the third and fifth Sustainable Development Goals (SDGs) targets: 3.1, 3.7, 3.8, and 5.6 [13, 14]. One of the key barriers to the availability and accessibility of family planning services in sub-Saharan Africa is the critical dearth of qualified health care providers. On the one hand, while reaffirming that human resources is at the core of each health care system around the world, the health workforce remains inequitably distributed in most sub-Saharan African countries, with rural areas suffering chronic and severe shortages of competent health care providers [15, 16]. On the other hand, lack of motivation and absenteeism of health care providers in impoverished countries widens the gap in quality family planning services [17]. In the bid to assuage human resource shortages, many countries have started to train less experienced health workers perform tasks that should otherwise be performed by qualified doctors or other highly-trained healthcare workers [18].

The World Health Organization (WHO), like many other stakeholders, recognise task sharing as a promising strategy to address the serious lack of health care workers to provide reproductive, maternal and new-born care in less wealthy countries [19–21]. By definition, task sharing involves the safe expansion of tasks and procedures that are usually performed by higher-level staff (i.e. physicians) to lay- and mid-level healthcare professionals (i.e. midwives, nurses, and auxiliaries) [22]. In the same perspective, WHO recommends that midwives be empowered to provide all family planning services except tubal ligation and vasectomy (Box 1). Also, initiation and maintenance of injectable contraceptives (standard syringe) can be performed by auxiliary nurses. Following WHO recommendations on *“Optimizing the roles of health personnel through the delegation of tasks to improve access to maternal and new-born health interventions”* (2012), regions including the Regional Office for Africa have started to mobilise local efforts with an aim to initiate and expand task sharing policies for family planning across respective member countries.

For the above reason, WHO Regional Office for Africa, in partnership with member countries and other key players such as the Ouagadougou Partnership for Family Planning Coordination Unit (UCPO), the West Africa Health Organisation (WAHO), and the United Nations Population Fund (UNFPA), organized a regional consultation meeting on task sharing in September 2016 with the aim of aiding nine pilot countries in developing action plans for the implementation of task sharing recommendations. Moreover, WHO Regional Office for Africa conducted an intensive advocacy which yielded a special resolution relating to task sharing for family planning endorsed by governments of the Economic Community of West African States (ECOWAS) region. In December 2019, a second regional advocacy meeting was held to expand the task sharing policies to an additional 11 English-speaking countries.

Four years after the first advocacy meeting, this paper explores the lessons learnt in relation to task sharing for family planning in five countries in the WHO African region. Specifically, the paper documents the status of task sharing for family planning policy implementation, its effect in coverage and use of family planning services, gauges key achievements, enablers and challenges to form a basis for the implementation monitoring and planning of task sharing initiatives for family planning in the region.

Box 1 Table of guideline recommendations for task sharing of contraception

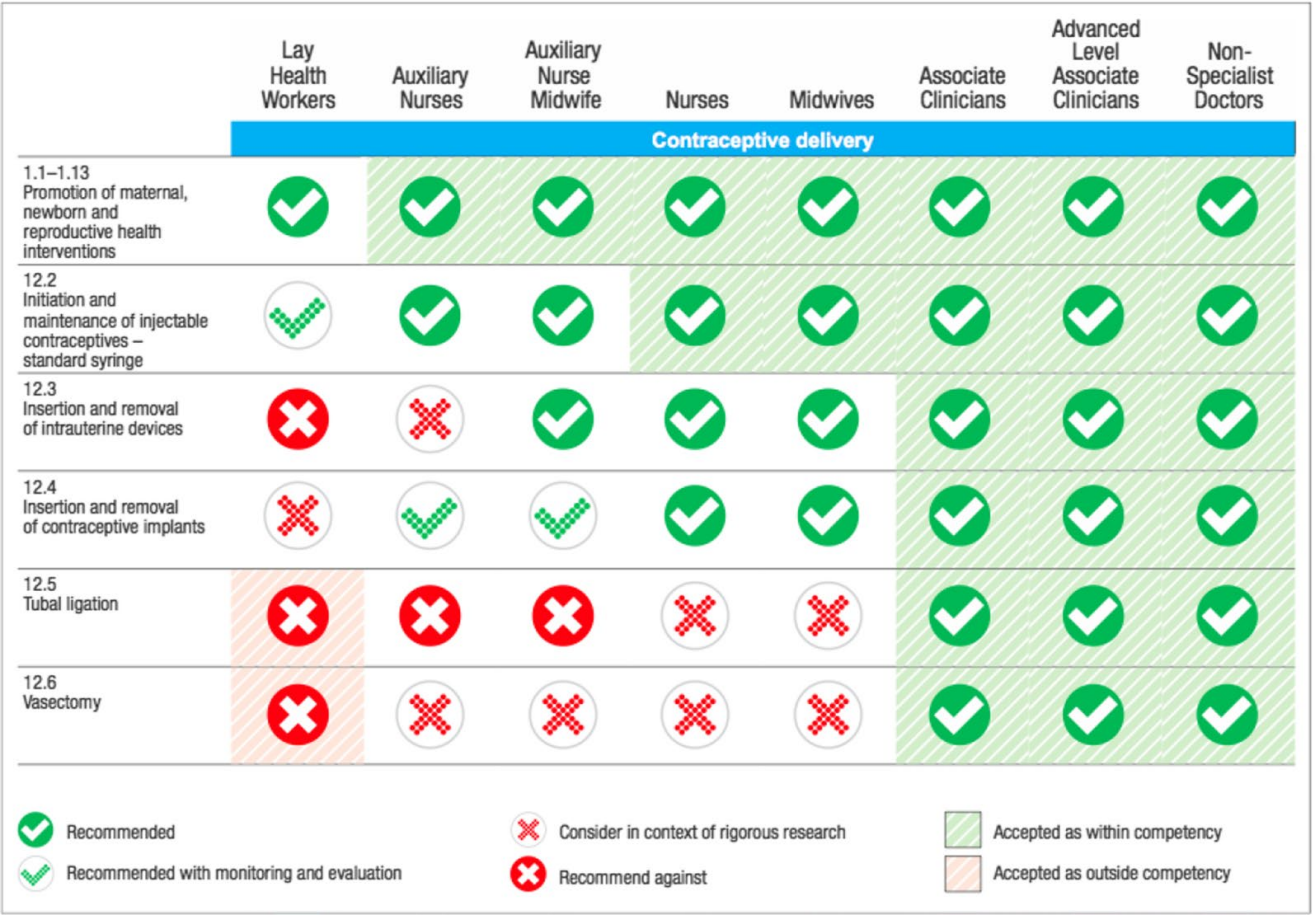

## Methods

The study applied the Rapid Programme Review (RPR) methodology to generate evidence on what WHO Regional Office for Africa and member countries can do to build on successes and tackle challenges with an aim to scale-up task sharing programmes for family planning region-wide. A rapid review is a knowledge synthesis method in which components of the systematic review process are simplified or omitted to produce information in a short period of time [23]. A RPR focuses on synthesizing information regarding a programme (task sharing programme for family planning in this case) through desk-review of programme documents, reports and key stakeholder information. The RPR methodology generates strong evidence and saves both time and costs, rather than conducting full programme reviews which are time-consuming and effort-intensive [24]. The method allows a rapid and progressive learning with conscious exploration and flexible use of methods without following a blueprint programme [25]. The review triangulated data from secondary sources with information from key informants in four countries which have already piloted the task sharing programmes for family planning. A trend analysis was done alongside an overview of system-level implementation enablers and barriers to successful implementation of task sharing programmes in the African context.

### Data collection

Data was collected in two steps. In the first instance, data for the RPR were obtained through a desk review of country task sharing for family planning policy documents, relevant implementation plans and guidelines, and annual sexual and reproductive health programme reports. In addition, data presented during the second Africa regional meeting on task sharing for family planning organised by WHO Regional Office for Africa was exploited to supplement document reviews. During this meeting, five countries which are piloting or implementing programmes on task sharing for family planning (Burkina Faso, Cote d’Ivoire, Ethiopia, Ghana, and Nigeria) presented success stories as well as challenges, lessons learnt and ways forward. A full list of countries that participated in the meeting is provided in Box 2.

In the second instance, WHO country offices were contacted to identify and obtain key informants on task sharing for family planning programmes in the five aforementioned countries. Through written communication (electronic mails), National Focal Points (NFPs) on sexual and reproductive health provided information on the country background, intervention packages, intervention impact, system-level enablers and challenges, and information on ways forward.

The country background helped to understand the baseline picture. Specifically, we collected information on the date when the first task sharing programme was piloted, the rollout process and, most importantly, the significant baseline family planning indicators. A list of full family planning indicators for Burkina Faso before (2010) and after (2019) implementation of the task sharing for family planning programme is shown in Table 1. Secondly, data on the type of task sharing intervention packages were collected. In addition, geographical reach and the type of tasks and healthcare professionals involved were documented. If available and applicable, an illustrative picture was also shared to demonstrate lay- and auxiliary-level cadres performing family planning tasks previously performed by higher healthcare professionals. Thirdly, we used Table 1 and collected data on key family planning indicators during the period of implementation of task sharing for family planning. Given the availability of enough data-points, baseline and midterm data were used to trace an indicator trend line. We also documented system-level levers and challenges that played an important role in the successful/unsuccessful implementation of task sharing programmes. This information is necessary for policymakers amid the aim by WHO Regional Office for Africa and member states of rolling-out and expanding task sharing for family planning programmes region-wide. Lastly, each country provided information on the next steps with concrete actions to be undertaken in the near future with regards to task sharing for family planning.

**Table 1:** Indicators before and during the task sharing pilot programme in Burkina Faso

Indicator	Year		% increase
	2010	2019	
Women of reproductive age (n)	3,812,595	4,693,950	23.12
Expected pregnancies (n)	881,489	1,118,519	26.89
Pregnant women (n)	734,574	989,245	34.67
Maternal mortality ratio	341	330	− 3.23
Total fertility rate	6.0	5.4	− 10.00
Fertility rate per woman of childbearing age (expressed per 1000 women aged 15–49)	206	173	− 15.58
Contraceptive prevalence (%)	15	30.70	104.67
Number of couples (husband-wife) using family planning services (n)	476,087	1,348,692	183.29
HIV prevalence among women of reproductive age (%)	1.00	0.70	− 30.00
National health budget (as per of total total)	8.90%	10.95%	23.03
Family planning expenditure per total health expenditure	18.10%	37.20%	105.52
Family planning expenditure per total sexual and reproductive health expenditure	7.50%	9.90%	32.00

Box 2. List of countries that participated to the WHO regional meeting on task sharing for family planning, 16–19 December 2019

1. Benin
2. Burkina Faso*
3. Cote d’Ivoire*
4. Ethiopia*
5. Ghana*
6. Guinea
7. Liberia
8. Malawi
9. Mali
10. Mauritania
11. Niger
12. Nigeria*
13. Rwanda
14. Senegal
15. South Africa
16. Tanzania
17. Togo
18. Uganda
19. Zambia

* Countries piloting or implementing programmes on task sharing for family planning

### Data analysis

Data was analysed in two steps. Step one consisted of compiling information from the country background, the task sharing intervention packages, the system-level enablers and challenges, and the ways forward. All data sources were verified to ensure reliability of reported information. In the event of missing data, a request was resent to the respective NFP who was asked to provide feedback within two weeks. Beyond a period of two weeks, the data was confirmed as “missing information”. For example, Cote d’Ivoire was excluded from analysis due to substantial missing data. Step Two consisted of a trend analysis of key family planning indicators. Owing to the limited number of data-points (often only two data-points), a trend line was only possible for Ghana and Nigeria. For Burkina Faso and Ethiopia, we compared proportions before and during task sharing interventions.

## Results

Results are mainly presented as text boxes of country overviews. In each box, we summarised findings on the country background, described existing task sharing intervention packages, quantified midterm programme impact, analysed system-level enablers and barriers, and suggested ways forward.

Box 3. Burkina Faso **Background**

Burkina Faso is a West African country struggling with severe health workforce shortages [26]. On average there is less than one physician per 10,000 people and 2.39 midwives per 10,000 people [27]. This is way lesser than the WHO doctor-population ratio of 1:1000. Burkina Faso piloted the first task sharing programme between 2015 and 2016 across 17,688 villages in the Hauts Bassin region, Boucle de Mouhoun region, Central West region, and Central region. Each participating village received two community health workers (CHWs) trained to provide injectable contraception (Sayana press) in addition to contraceptive pills. Task sharing was also piloted at health facility level where midwives were trained to perform long acting reversible contraception (LARC) procedures—the intrauterine contraceptive device (IUCD) and implants. Evaluation of the pilot programme yielded promising results, leading to the validation and nationwide rollout of the task sharing programme in November 2017.

**Task sharing intervention packages**

CHWs and midwives received comprehensive specific training needed for performing new family planning tasks. The training was provided by the Ministry of Health. There were also adequate post-training follow-up and monitoring of the health workers. Furthermore, in addition to an effective supply chain of services, mechanisms for quality control were put in place. Additionally, advocacy meetings and community mass mobilisation campaigns were regularly conducted. Joint-field monitoring and evaluation missions were conducted to enable an early detection of potential enabling factors and challenges that affect the successful programme implementation. Results were disseminated through regional and sub-regional meetings in Burkina Faso, Ghana, Kenya, and Cote d’Ivoire.

**Impact**

Partial results showed an increase in new users. For instance, within a period of six months (February to September 2017) during the implementation of task sharing for family planning by trained CHWs and midwives, a total 1225 implants—of which 857 were new users—were administered. A total of 384 IUCDs, of which 238 were new users, were provided by newly trained midwives. In the same period, CHWs provided 3541 injectable contraceptives (Medroxyprogesterone acetate)—of which 1013 were new users— and 1257 contraceptive pills, of which 241 were new users. Other family planning indicators are presented in Table 1.

**Enablers**

It stood out that the strong commitment and stewardship of health authorities from the top to the bottom levels, the expansion of contraceptive options, the community involvement, and the improved financial and geographic accessibility of family planning services played an important facilitating role.

**Challenges**

Notable challenges included data reporting, as routine paper-based reporting system was solely used, and financial constraints. Also, the programme was fraught with insufficient funding causing great irregularities in the payment of CHWs incentives. Evidence has confirmed that such a financial challenge has potential for reducing provider motivation [28, 29].

**Ways forward**

A commitment maker since 2012 and a member of both the Ouagadougou Partnership and SWEDD (Sahel Women’s Empowerment and Demographic Dividend project), Burkina Faso has taken its FP2020 commitment seriously through its 2017–2020 RH/FP strategy, which incorporates task sharing for family planning [30]. Burkina Faso vowed to build on successes to strengthen task sharing programmes through the recruitment and training of lay- and auxiliary-level healthcare providers by the Ministry of Health. Furthermore, there is a robust financial pledge and advocacy from political and administrative authorities, technical and financial partners and non-governmental associations working in the field of family planning.

On the one hand, time comparison shows an increase in the number of women of reproductive age and that of expected and real pregnancies. On the other hand, there has been a decrease in fertility rate and maternal mortality ratio. Overall, Burkina Faso showed promising results for family planning services. Key improvement features of family planning include an increase in contraceptive prevalence which more than doubled (105% increase), the increase in numbers of couples using a contraceptive method which nearly tripled (183% increase), and an increase in family planning expenditures. Moreover, in 2019, family planning averted 11.56% of expected pregnancies (comparison of expected and real pregnancies in 2019).

Box 4. Ethiopia **Background**

Ethiopia has a total population of 100 million people of whom 83.6% live in rural areas [31]. Majority of Ethiopia’s population is made up of young people, with 45% representing those under 15 years old and 71% under 30 years old [32]. Women of reproductive age account for 24% of Ethiopia’s population [31]. Each year, Ethiopia expects a total number of 3 million pregnancies. The country’s population growth rate is 2.6% per year and the total fertility rate is 2.3 births per woman in urban settings and 5.2 births in rural areas [31].

**Task sharing intervention packages**

In Ethiopia, the task sharing programme was piloted in three different phases. Phase one involved the Implanon programme which was first piloted in 8 districts in 2009. Provision of Implanon was shifted from healthcare facility level to community level. Phase Two, which started in 2011, was the IUCD task sharing programme which was piloted in one region where the device was inserted and removed by midwives and nurses rather than physicians. Phase Three was where the IUCD provision was further lowered to auxiliary nurses in 2016 across 66 selected health posts.

**Impact**

Within a period of 12 months (from July 2018 to June 2019), 1.43 million clients received a LARC method (1,362,149 women received Implanon and 64,073 women received IUCD). Moreover, contraceptive prevalence rate has doubled every five years from 2000 (CPR = 6.1%) to 2019 (CPR = 41%). Another supporting point is the decline in total fertility rate which fell from 6.0 to 4.6 in the same period. Similarly, unmet contraceptive needs were higher in 2011 (25.3% of unmet contraceptive needs) as compared to 2019 (22% of unmet contraceptive needs). Equally important, IUCD utilization rate increased from less than 2% in 2011 to more than 11% in 2019.

**Enablers**

The successful implementation of Ethiopia’s task sharing pilot programme was a result of political commitment. There has been a visible political will and support by the Government of Ethiopia. Specifically, the Government signed international family planning policies, elaborated national policies and strategies in support of the implementation of family planning standards, promoted and stimulated demand for family planning services, and continuously increased the overall health budget over the past decade.

**Challenges**

Key challenges included lack of awareness and misconceptions regarding some contraception methods such as long-acting family planning (LAFP) methods among the target population, shortages of medical equipment and logistics, poor infrastructure (electricity, water, and roads), and poor mentorship and supporting supervision.

**Ways forward**

Looking ahead, Ethiopia aims to accelerate strategies to increase the demand for family planning services until the very remote communities, to enhance service availability and accessibility, to improve provider competency and performance, and to strengthen mentorship and supportive supervision. Ethiopia has committed to the FP2020 call to action that urges global health and development partners to adopt task sharing as a key solution for increasing access to contraception [33].

Box 5. Ghana **Background**

Despite Ghana having halved the maternal mortality ratio in the past 20 years (760 maternal deaths per 100,000 live births in 1990s versus 310 maternal deaths per 100,000 live births in 2019), the country still has one of the lowest contraceptive prevalence rates in the region (22.2% in 2014) [34]. In this country, task sharing programmes for family planning started in 2008 when 33 Community Health Nurses (CHNs) from 6 regions received training in Jadelle insertion and removal. After the Jadelle programme, nurses and midwives from across Ghana started receiving tailored training on Implant provision nationally in 2013.

**Task sharing intervention packages**

From 2013, Implants were inserted and removed by CHNs, nurses, and midwives.

**Impact**

**Fig. 1:**
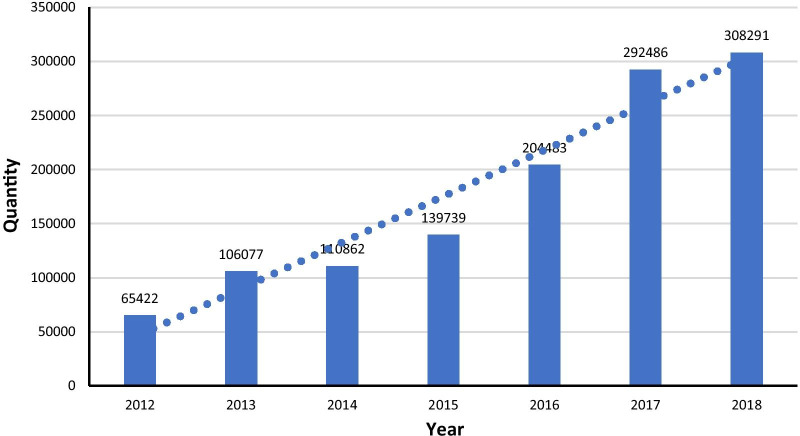
Trend in implant utilisation in Ghana (Source: Ghana Maternal Health Survey 2017. 2018)

Implant users increased from 11 users per provider in 2013 to 18 users per provider as of 2018 (DHIMS2). Contraception prevalence rate among married women increased from 18.6% in 2008 to 19.8% in 2014 among rural residents and from 15.1% in 2008 to 24.6% in 2014 among urban women. The trend in Implants (Jadelle and Implanon) utilisation is depicted in Fig. 1 below. As can be seen, the number of Implant users tripled from the onset of countrywide task sharing programme (2013) to 2018.

**Enablers**

Like for other countries, levers of success included the political will and commitment of Ghanaian Leaders, concerted advocacy programmes, stakeholder involvement, quality monitoring and supervision, and support by regional resource teams.

**Challenges**

A couple of notable challenges concerned funding gaps and the uneven distribution of CHNs in task sharing.

**Ways forward**

Ghana’s main next step in task sharing for family planning is to initiate a Midwifery Assistant programme. This programme will enable the training of CHNs as “Midwifery Assistants” who will be sent across the country. Following the training, Ghana projects to further select and train 72 Midwifery Assistants on IUCD in a one-year pilot programme followed by an evaluation and countrywide rollout. Furthermore, as part of its commitment to the FP2020 targets to increase the number of women and girls using modern contraception from 1.5 million to 1.9 million by improved access to and availability of quality family planning services, Ghana aims to support capacity building of Community Health Nurses through task sharing of LARC provision to strengthen the provision of FP services nationally [35].

Box 6. Nigeria **Background**

Nigeria has an estimated population of 200 million with 45 million women of reproductive age [36]. The total fertility rate is 5.3 with current contraceptive prevalence rate of 12%. While unmet need for contraception was 19% in 2018, the country aims to attain 27% of contraceptive coverage by 2020 [36]. The Federal Government of Nigeria passed the task sharing policy in 2014 through which Community Health Extension Workers (CHEWs) received training on LARC and a subsequent authorisation to provide and remove Implants and IUCD.

**Task sharing intervention packages**

From 2014, nurses, midwives, and CHEWs became responsible for the provision of the entire family planning arsenal except tubal ligation and vasectomy (Box 1). They provided family planning counselling and education, promoted dual protection for HIV positive women, inserted and removed Implants and IUCD, and provided injectable contraception.

**Impact**

**Fig. 2:**
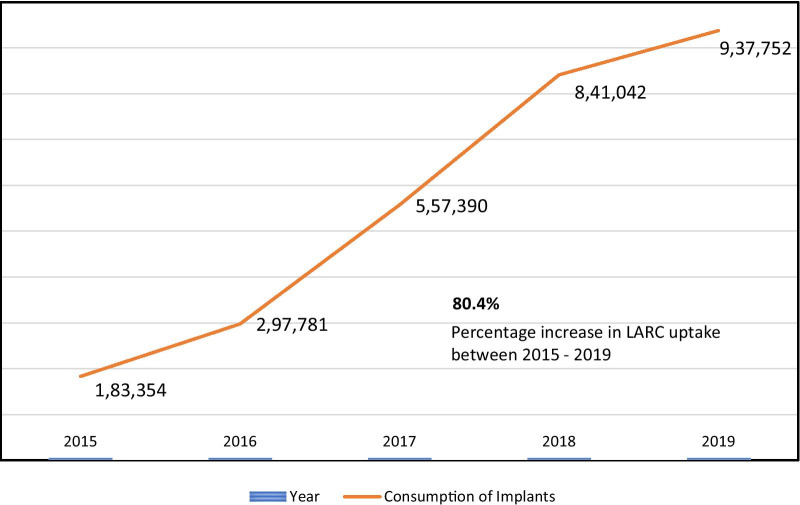
Trend in implants uptake in Nigeria (Source: Nigeria Demographic and Health Survey 2018)

Implementation of the task sharing policy for family planning increased the uptake of LARC. Figure 2 illustrates the uptake in implants increased by 80% within a period of four years (2015 to 2019).

**Enablers**

In Nigeria, there was collaboration with professional bodies which enhanced acceptance and ownership of task sharing programmes. Another lever was the ability of majority of CHEWs to fast-learn and absorb training materials.

**Challenges**

Adverse circumstances were limited to a small number of poor-performing family planning providers. Also, the programme required intense follow-ups and mentoring which meant that it became costly despite benefits outweighing the costs.

**Ways forward**

Preliminary results were a powerful tool to advocate for the scaling up of task sharing for family planning programmes. Therefore, from 2019, Nigeria embarked on rolling out the pilot project at country level. As part of their FP2020 commitments, Nigeria plans to train at least 3700 community health workers (CHWs) for the delivery of LARC and support task shifting so CHWs in rural areas can provide multiple methods [37].

## Discussion

This rapid review set out to identify lessons learnt from the task sharing for family planning pilot programmes in four African countries with an aim to assist WHO Regional Office for Africa in identifying areas and strategies to strengthen advocacy for policy expansion region-wide. Data was collected, analysed, and presented according to five subdomains: country background, task sharing intervention packages, impact, enablers and challenges, and ways forward.

The findings of this review confirm that African countries share a similar background characterised by higher fertility and population growth rates, younger and mostly rural populations, lower contraceptive coverage rates, higher rates of unmet needs for contraception, severe human resource shortages with existing health workforce being unevenly and inequitably distributed; among others. This population trend, which hinders the attainment of development and health goals in Africa, has existed for more than two decades and will continue to rise unless substantial changes are made [38–40].

Common task sharing interventions involved CHWs, midwives, and nurses. There may have been different naming based on country-specific contexts, but they all referred to the above three categories of healthcare providers. For all countries studied, CHWs, midwives, and nurses were trained on the provision of contraceptive pills and LARC namely, Implants and IUCD. Based on WHO recommendations in Box 1, however, it is not recommended for lay-level health workers to insert and remove IUCD. Likewise, Auxiliary nurses are not allowed to insert and remove IUCD unless in the context of rigorous research (Box 1). Unfortunately, we did not obtain data on adverse effects that could have resulted from CHWs and midwives inserting and removing IUCD. Although this may be true, previous studies from the African context did not report side effects or incidents from CHWs providing LARC namely IUCD. Instead, CHWs increased uptake of IUCD utilisation in Rwanda [41] and in Ethiopia [42]. This evidence corroborates our findings.

Our findings indicated an increase in family planning indicators resulting from the task sharing programmes. In Burkina Faso, LARC uptake increased by greater than three times within a period of six months with 232.9% new implant users and 163.0% new IUCD users. There was a slower uptake for Depo-Provera and contraceptive pills with 40.1% and 23.7% of new users, respectively. Most importantly, the new contraception programme averted 11.7% of expected pregnancies in 2019. In Ethiopia, results from this study showed a doubling contraceptive prevalence rate with declining rates of total fertility and unmet needs for contraception. In Ghana and Nigeria, there has been an increase in the number of new users with a significant uptake of Implants and IUCDs. Similar results have been found in many other African contexts. For instance, the Democratic Republic of Congo (DRC) is one of the countries that have suffered the most from human resource shortages in the whole world. A new task sharing programme that sought to promote LARC in remote areas was able to achieve 38,662 new users within a period of 5 years [43].

To summarise, despite countries being at different stages in terms of promotion and implementation of task sharing policies, they have some achievements in common. These include the presence of policies, regulations, or laws on task sharing; the presence of community health strategies and programmes, ongoing dialogues and discussions on task sharing, in-country communication strategies and governmental support. Countries also share some common challenges mainly the difficulties in retention of lower cadres due to financial constraints (incentives), inadequate documentation of successful processes to support internal learning and external lessons sharing, and difficulties capturing data on service provision. Moreover, they share common priorities: advocacy, capacity building, and financial pledge for impact sustainability.

## Conclusions

Task sharing is important to ensuring that everyone has access to family planning services they need to space or limit childbearing. Task sharing for family planning should be contextualised to align with country situations. Furthermore, training and monitoring of lay- and auxiliary-level cadres remains a dire necessity. Country plans for task sharing for family planning should be positioned within the broader national objectives of Universal Health Coverage (UHC) and Primary Health Care (PHC) in order to achieve the SDGs agenda. Plans should be specific on and include documented best practices and promote mentoring (i.e. through South-South learning) as a viable solution to support the advancement of best practices. Evidence from the present review point to possible association between task sharing for family planning and increased contraceptive uptake, which makes task sharing a potential viable intervention. It is against this evidence that we recommend WHO Regional Office for Africa and member states to build on the evidence from Burkina Faso, Ethiopia, Ghana, and Nigeria in elaborating country policies for task sharing in family planning.

## Limitations

The small sample size of key informants who provided information to the RPR could be considered a limitation to the study. Furthermore, the collection of electronic information rather than verbal could have limited the depth of information provided. However, current and available documents on task sharing for family planning ably supplemented the information provided. It is important to mention that attribution of the family planning outcomes to the task shifting intervention should be handled with caution as the RPR cannot be used in place of causal studies. Therefore, we recommend additional studies that can statistically attribute outcomes to the task sharing intervention.

## Data Availability

Data and materials used for this review are available either online (policies and country reports) or from the corresponding author (meeting presentations, information from key informant interviews).

## Abbreviations

WHO: World Health Organization
SSA: Sub-Saharan Africa
RPR: Rapid programme Review
LARC: Long-acting reversible contraceptives
LMICs: Low- and middle-income countries
UCPO: Ouagadougou Partnership for Family Planning Coordination Unit
UNFPA: United Nations Population Fund
ECOWAS: Economic Community of West African States
NFPs: National Focal Points
CHWs: Community health workers
IUD: Intra-utérine device
IUCD: Intra-utérine contraceptive device
HIV: Human Immunodeficiency virus
LAFP: Long-acting family planning
SDGs: Sustainable Development Goals
CHEWs: Community Health Extension Workers
CHN: Community Health Nurse
